# Breast magnetic resonance imaging: tips for the diagnosis of silicone-induced granuloma of a breast implant capsule (SIGBIC)

**DOI:** 10.1007/s13244-017-0564-3

**Published:** 2017-07-14

**Authors:** Eduardo de Faria Castro Fleury, Ana Claudia Gianini, Veronica Ayres, Luciana C. Ramalho, Rodrigo Oliveira Seleti, Decio Roveda

**Affiliations:** 1 Department of Radiology, Santa Casa de Misericórdia de São Paulo, Rua Maestro Chiaffarelli, 409, Jardim Paulista, São Paulo, SP 01432-030 Brazil; 2 Department of Radiology, IBCC-Insituto Brasileiro de Controle do Câncer, São Paulo, SP Brazil

**Keywords:** Breast implant, Silicones; granuloma, Magnetic resonance imaging, Mass, Breast neoplasm

## Abstract

Complications resulting from the placement of silicone breast implants are becoming more frequent in our clinical practice. This is due to the increase in breast aesthetic surgeries at the beginning of the century, where breast augmentation using silicone implants was the main intervention performed. Generally, studies that discuss the complications of breast implants are restricted to reports of intra- or extra-capsular ruptures, contractures and haematomas. Currently, much importance has been given to anaplastic large cell lymphoma (ALCL) as a more severe complication related to silicone implants. Recently, granuloma formation induced by silicone particle bleeding from intact breast implants has been described when the free silicone comes into contact with the fibrous capsule of the implant. Few studies have demonstrated the characteristics and diagnostic keys for this entity. The objective of this study is to present cases of SIGBIC diagnosed in our service and to discuss the main findings that allow its diagnosis.

*Teaching Points*

• *Breast implants induce fibrous capsule formation at the periphery of the implant.*

• *Gel bleeding is inherent in all types of silicone breast implants.*

• *Gel bleeding induces silicone-induced granuloma of breast implants.*

• *Main diagnostic tips: heterogeneous mass, black-drop sign and late enhancement.*

## Introduction

In the early 2000s, an epidemic of breast augmentation surgeries was observed in Brazil. The economic boom experienced at the beginning of the century associated with socio-cultural factors led to this epidemic. Seventeen years later, some repercussions of these interventions are being observed in our society.

According to the International Society of Aesthetic Surgery (ISAP), Brazil has the second highest number of aesthetic surgeries worldwide, second only to the USA. It is estimated that in 2015, 1.22 million aesthetic surgeries were performed. Of these, breast augmentation with the placement of breast implants was the second most performed surgery, the first being liposuction. In that year approximately 158,950 breast implants were placed in Brazilian women, the majority for aesthetic purposes. Silicone implants are the most used in our society [1].

The medical literature describes many complications related to the presence of silicone implants, the main ones being seromas, infections, haematomas, and intra- and extracapsular ruptures [2–4]. Anaplastic large cell lymphoma (ALCL) is also highlighted as a more severe complication. In the past year, the Federal Food and Drug Administration (FDA) of the USA has been concerned about the increased incidence of lethal cases of ALCL [5].

Recently, findings of silicone-induced granuloma have been reported in the breast implant capsule, termed silicone-induced granuloma of the breast implant capsule (SIGBIC) [6]. This finding was reported in a patient with intact silicone breast implants with clinical findings of capsular contracture submitted to diagnostic breast magnetic resonance imaging (MRI).

The purpose of this pictorial essay is to describe the mechanism of development of this granulomatous process and to demonstrate the main findings by magnetic resonance imaging. The findings are based on diagnostic breast MRI scans performed at our service. Informed consent was obtained for all patients. Approval by the institutional ethics committee was waived because of the nature of the manuscript.

## Pathophysiology of SIGBIC

To understand the formation of GISGBIC, it is important to know the pathophysiology involved in its formation process. Some concepts are of extreme importance for understanding this process, such as knowing the elements that participate in granuloma formation, the phenomenon of gel bleeding in intact breast implants and autoimmune reactions to silicone-content particles.

Placement of the breast implant, a non-biological material, in the breast induces fibrous capsule formation at the periphery of the implant. The cell composition of the capsule has been extensively studied, and the results point to a role of the immune system in the pathologies developed in the capsule. The predominant cell types reported within the fibrous capsule are macrophages, lymphocytes and fibroblasts. Mast cells are also reported, which can express renin, histamine and tumour growth factor β-1 (TGF-β). It is supposed that the mast cells activate fibroblasts, which produce collagen, one of the mechanisms of capsule contracture. A role of T cells has also been hypothesised. The fibrous capsule works as a physical barrier that isolates the intra-capsular contents from the rest of the body [7].

The phenomenon of gel bleeding is inherent in all types or models of silicone breast implants, which are soft, round or cohesive with anatomical shape. Even the most modern implants that contain an extra outer layer of protection do not prevent silicone bleeding; it only slows down the process. The phenomenon of gel bleeding determines the weakening of the elastomer independent of the brand. This is supposed be the triggering factor for implant rupture [8].

**Fig. 1:**
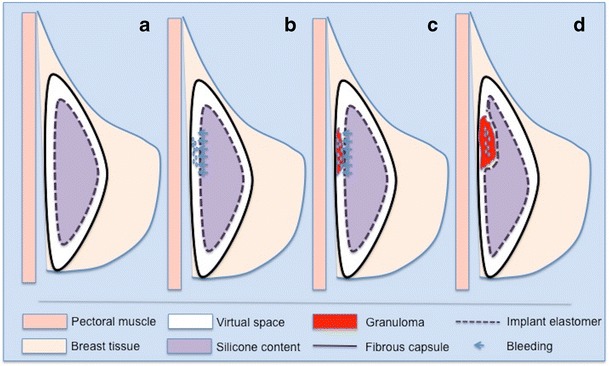
Scheme of silicone-induced granuloma formation (**a**, **b**, **c**, **d**). Intact implant (**a**). Gel bleeding to the virtual space (**b**). Granuloma formation by macrophages (**c**). Expansive effect of granuloma (**d**)

There is a virtual space between the breast implant and the fibrous capsule, where theoretically there should be no structure. Figure 1 schematically shows the granuloma formation induced by silicone bleeding from an intact breast implant in the fibrous capsule (Fig. 1) [6].

Tervaert and Kappel [9] described the association between autoimmunity and silicone exposure as follows:

- Contact of silicone-content particles bleeding from the intact breast implant with the fibrous capsule;
- Capture of these silicone-content particles by macrophages with entrapment within the lysosomes, activating the macrophages;
- Macrophage apoptosis by the production of cytokines [interleukin-1b, reactive oxygen species (ROS) and reactive nitrogen species]. It releases silicone-content particles, which are captured by another macrophage, generating a vicious circle;
- Massive production of interleukin-17 with neutrophil influx activation and ROS production;
- Myeloid granular enzyme (myeloperoxidase) release, which produces hypohalous acids; these have antimicrobial activity;
- There is also lymphocyte stimulation by a type 2 inflammatory response, increasing the levels of IgE and IgG1 associated with chronic activation of T lymphocytes. This chronic activation is probably due to the dysfunction of negative regulation and T cells;
- Finally, the process also activates fibroblasts, with production of collagen, and myofibroblasts, which promote capsular contracture [6, 8].

As there is a physical barrier between the rest of the body and the intracapsular space, the vascularisation inside the capsule is poor, which makes the process indolent.

## Clinical findings of SIGBIC

In 2015, Tervaert and Kappel reported that of the 600 patients evaluated in an ambulatory immunology clinic, 32 had silicone breast implants. All patients with silicone implants presented with silicone implant incompatibility syndrome (SIIS), whereas none of the patients with saline or mixed implants presented signs of autoimmune disease. The main symptoms associated with SIIS were capsular contracture and/or systemic manifestations such as fatigue, arthralgia, myalgia, asthenia and/or fever. Of the 32 patients, a systemic autoimmune disease was diagnosed in half of them. These patients might have had undiagnosed SIGBIC [9].

In this pictorial essay, all patients with SIGBIC presented with clinical signs of implant capsular contracture, characterised by hardening and pain in the compromised breast. These symptoms are usually interspersed by periods of spontaneous remission or by use of anti-inflammatory/corticosteroid therapy as reported by the patients. These findings suggest a relationship between silicone implants and autoimmune disease.

The time from implant placement surgery ranged from 2 to 20 years. The age of the patients ranged from 30 to 71 years. None of the patients had other chronic diseases.

Of the eight patients described in the study, three have had a history of repair surgery after breast cancer treatment and the other five of breast augmentation (aesthetic surgery). Six patients reported pruritus in the upper and/or lower extremities with beginning symptoms related to implant placement.

## Breast magnetic resonance imaging for breast implant evaluation

Magnetic resonance is the examination of choice for evaluating breast implants. Currently, it is mainly used to evaluate intra- or extra-capsular ruptures of breast implants. Usually a specific protocol is used for the evaluation of breast implants, with dedicated sequences that perform water and silicone suppression. Many services choose not to perform intravenous contrast for evaluation of implants. The absence of contrast media makes it impossible to distinguish SIGBIC from intra-capsular haematoma. Searching the bibliographic databases online on the internet, only a few references to this entity were found.

Our hospital is a regional reference centre for breast cancer diagnostics and treatment. Breast MRI scans correspond to about 15% of the MRI scans performed at our institution. Approximately 38% of the breast MRI indications at our service are for breast cancer screening in high-risk patients, while the other 62% are diagnostic scans as established by the Colégio Brasileiro de Radiologia (CBR): evaluation of breast implants, staging of breast carcinoma and/or evaluation of response to neoadjuvant therapy, and occult carcinoma screening [10].

Until 2015, the breast implant MRI protocol at our institution was performed exclusively without contrast media. We only added water and silicone saturation sequences to the standard protocol. However, in the cancer screening patient group with breast implants who underwent contrast dynamic sequences and who presented intracapsular seroma, we observed that some had a solid lesion instead of seroma, with late contrast enhancement. From this finding, we started to adopt the dynamic series for all patients with breast implants to our protocol.

Currently, about 32% of our patients who performed breast MRI scans have breast implants. Of them, approximately 56% have capsular contracture, and 12% have findings compatible with SIGBIC.

## Imaging findings of SIGBIC

Based on the cases of our clinical practice, we describe the tips to facilitate the diagnosis of SIGBIC. Among the MRI findings, we highlight an intra-capsular mass, black drop sign and late contrast enhancement. As associated findings, we note intra-capsular collection and capsular contracture (Table 1).

**Table 1:** Tips for diagnosising silicone-induced granuloma of a breast implant capsule (SIGBIC)

	Sign	MRI sequence	Tips
Main findings	Mass	T2 weighted	Heterogeneous high signal
	Black drop	Dynamic	Hyposignal focus at the interface between the mass and the prosthesis
	Enhancement	Dynamic	Late enhancement (4 min)
Associated findings	Fluid collection	Dynamic	No enhancement
	Contracture	Dynamic	Thickening and enhancement of the whole capsule

### Intracapsular mass

**Fig. 2:**
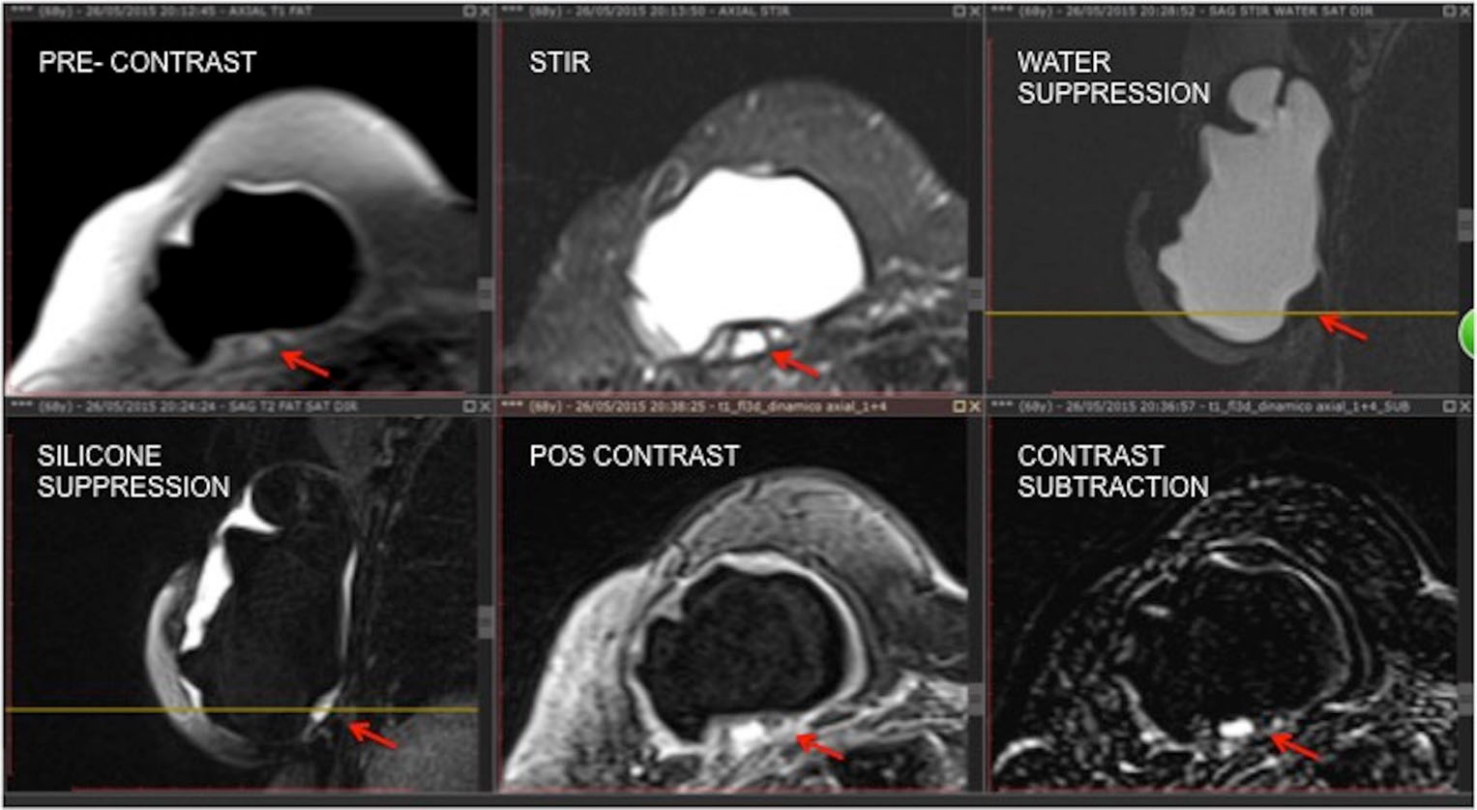
Mass effect of granuloma exerted on the breast implant (red arrow). The mass presents heterogeneous hypersignal at T2 (STIR) and hyposignal in the sequence of water suppression and pre-contrast. There is late enhancement after the dynamic phase. A 60-year-old female with breast implants for 3 years presenting clinical signs of contracture

**Fig. 3:**
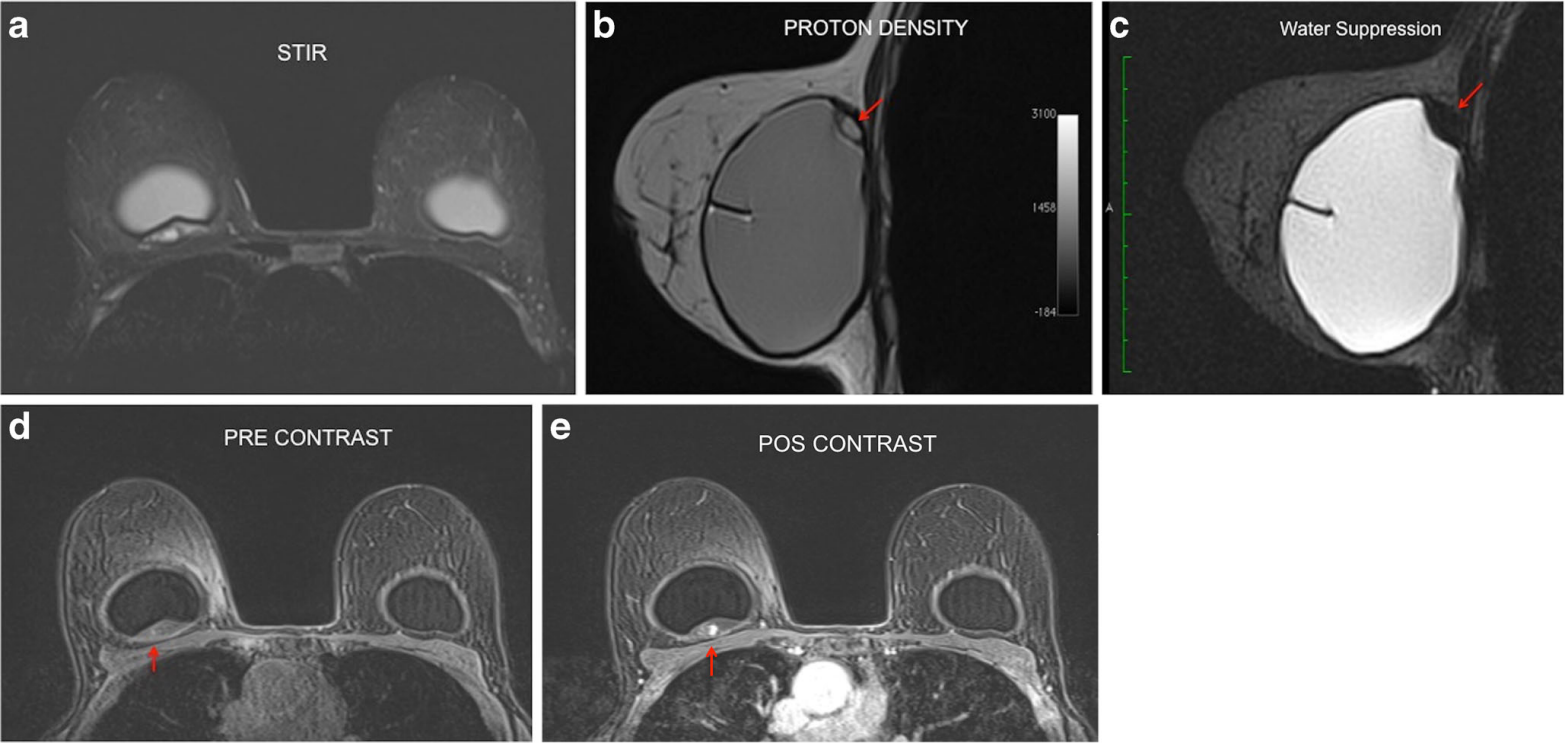
A 42-year-old female with inflammatory signs and tightening of the right breast (**a**, **b**, **c**, **d**). STIR sequence shows a heterogeneous lesion behind the prothesis (**a**). Proton density sequence confirms the location of the lesion (red arrow) (**b**). In the water suppression sequence, we emphasise the expansive effect exerted by the mass (red arrow) (**c**). Pre- and post-contrast dynamic phases demonstrate granuloma enhancement (red arrow) (**d**, **e**)

**Fig. 4:**
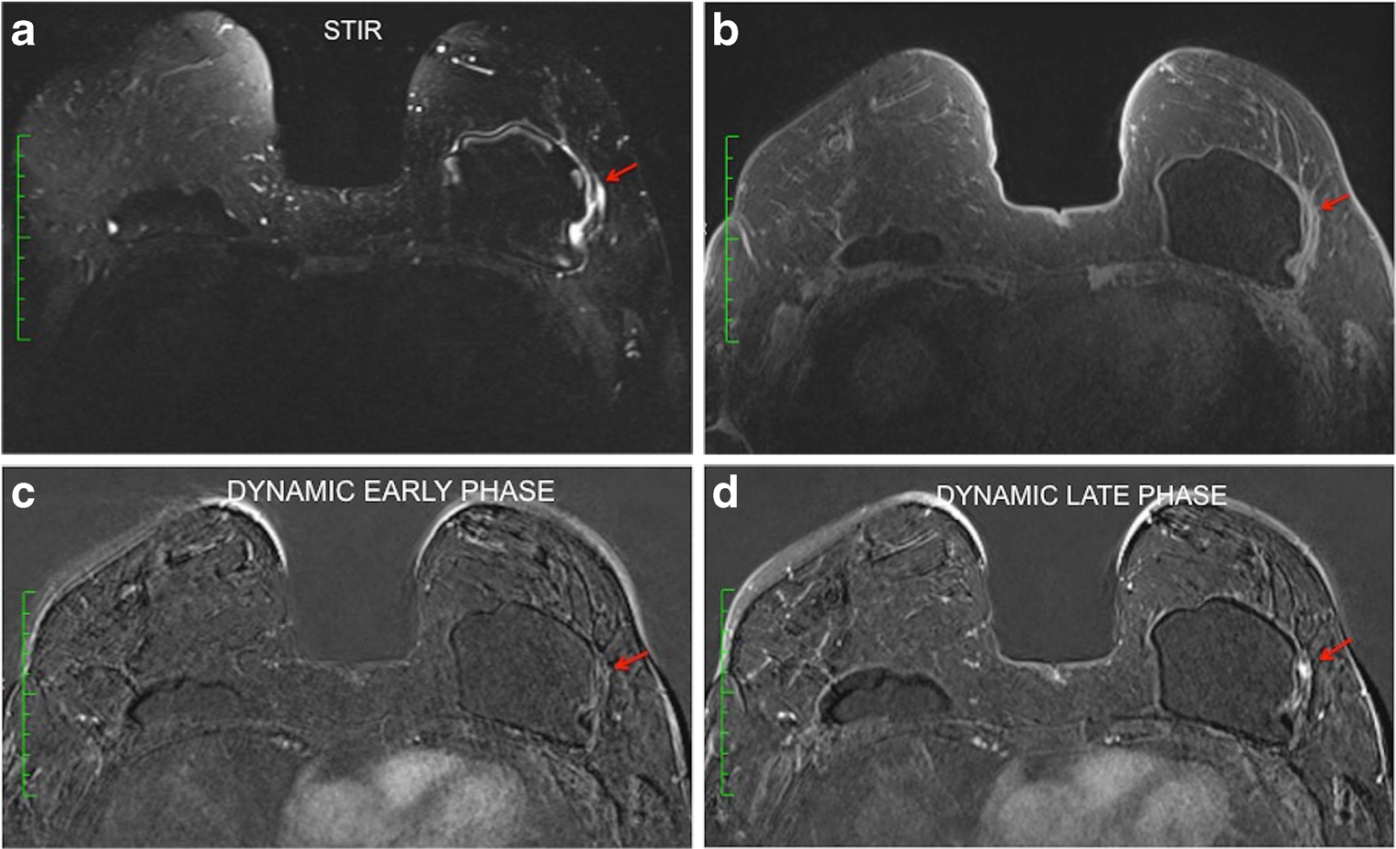
A 71-year-old female with suspected rupture of the left breast implant (**a**, **b**, **c**, **d**). Heterogeneous collection on the lateral side of the T2 implant (red arrow) (**a**). Sequences pre- and post-contrast with subtraction demonstrate the late enhancement pattern of the lesion (**b**, **c**, **d**)

**Fig. 5:**
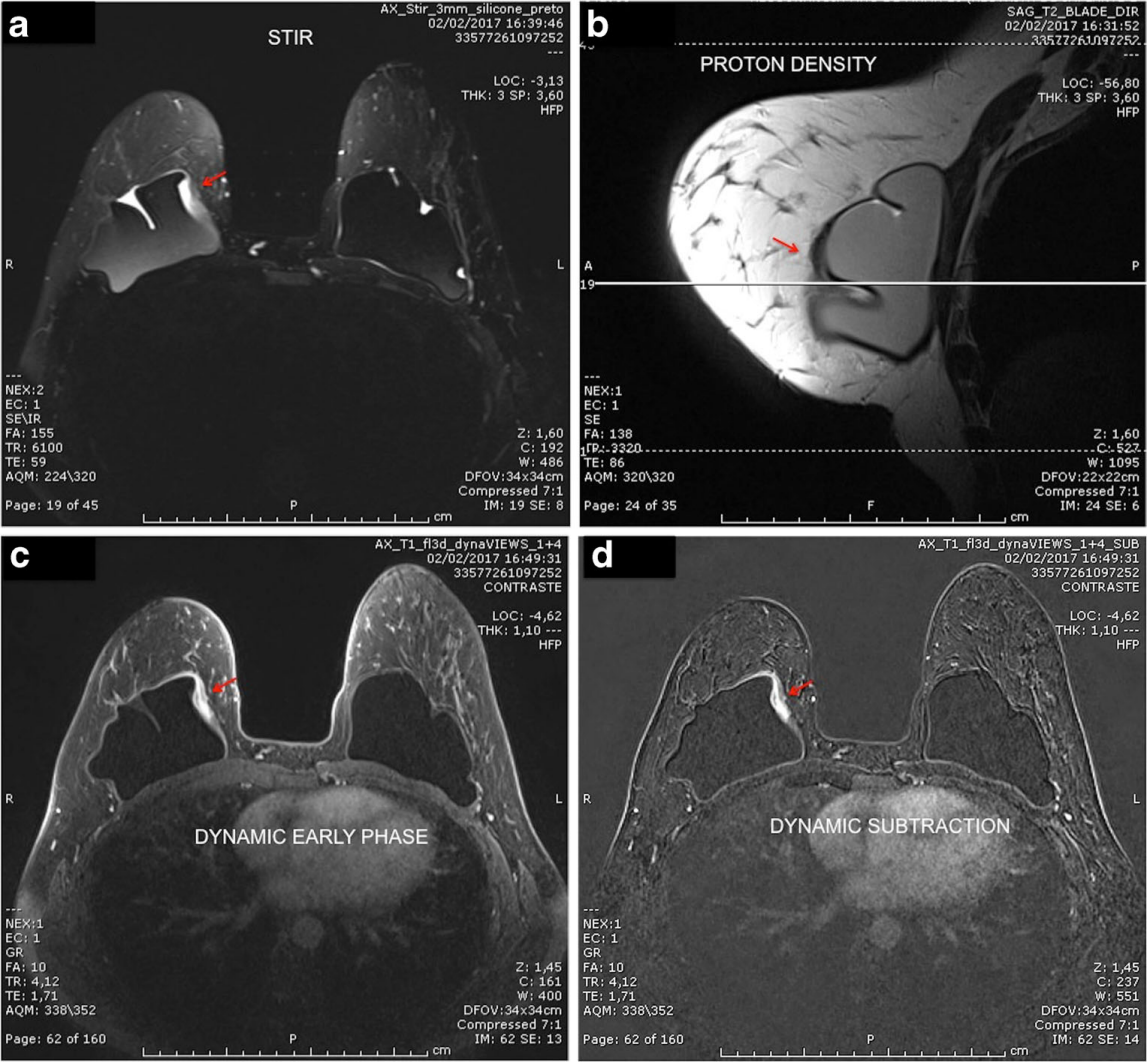
A 30-year-old female with stiffness on the medial aspect of the right breast, with inflammatory signs and pain (**a**, **b**, **c**, **d**). Heterogeneous mass on the medial aspect of the breast implant at STIR (red arrow) (**a**). Proton density sequence demonstrates a mass inside the implant fold (red arrow). The cross-reference line is used to correlate the multiplanar location of the lesion (**b**). Pre- and post-contrast dynamic phases demonstrate granuloma enhancement (red arrow) (**c**, **d**)

An intracapsular mass usually has a high heterogeneous signal in T2-weighted sequences and hyposignal in T1-weighted sequences. It is a slow-growing mass that has a compressive effect on the breast implant. Its main differential diagnosis is intracapsular haematoma, which makes the use of intravenous contrast essential (Figs. 2, 3, 4 and 5).

### Black drop sign

**Fig. 6:**
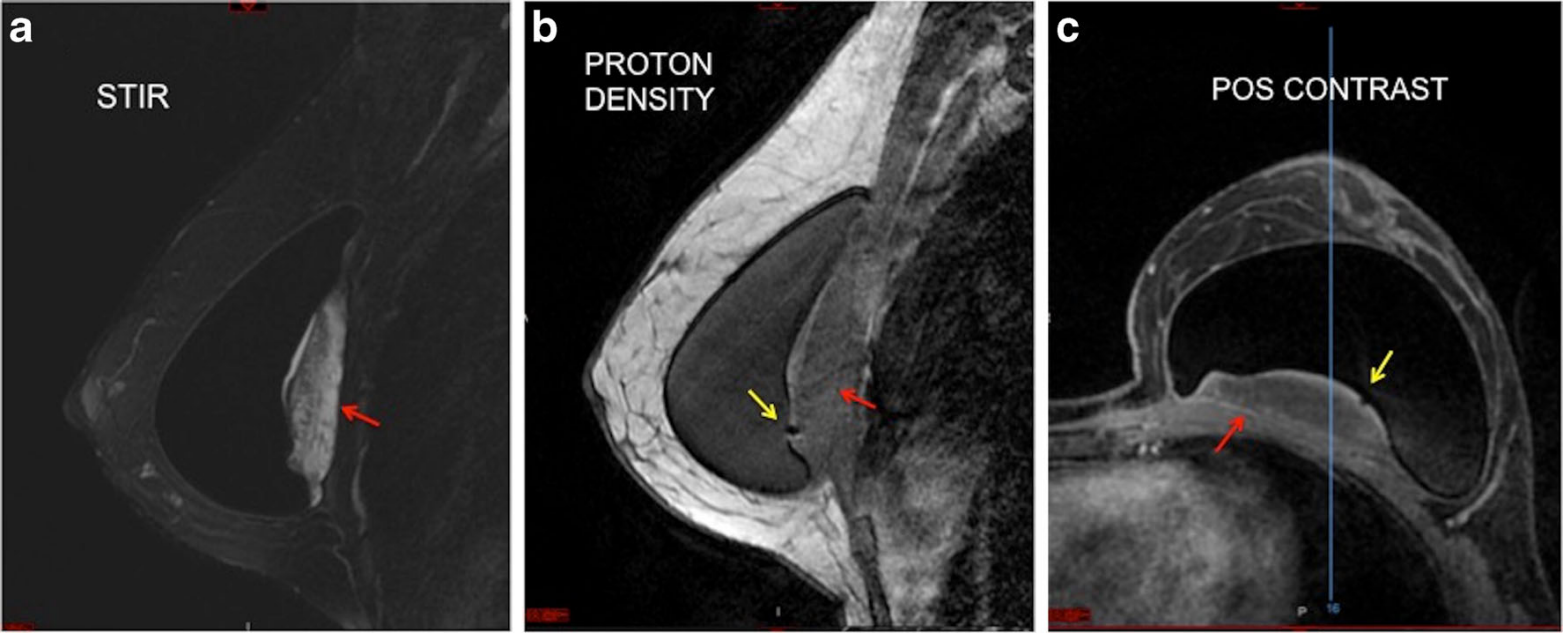
Black drop sign (**a**, **b**, **c**). Mass with a heterogeneous hypersignal posterior to the breast implant at STIR (red arrow) (**a**). Black signal focus (black drop sign) at the interface between the implant and the granuloma at proton density imaging (yellow arrow) (**b**). Post-contrast phase demonstrating the black drop sign (yellow arrow). We can see the late phase enhancement of the mass (red arrow) (**c**). A 38-year-old female with an implant for 2 years

This sign was found in all of our studied cases. It consists of a marked hyposignal focus at the interface between the breast implant elastomer and the granuloma on the dynamic sequence. This sign was visualised in the area of granuloma formation in all our cases (Fig. 6).

### Late contrast enhancement

**Fig. 7:**
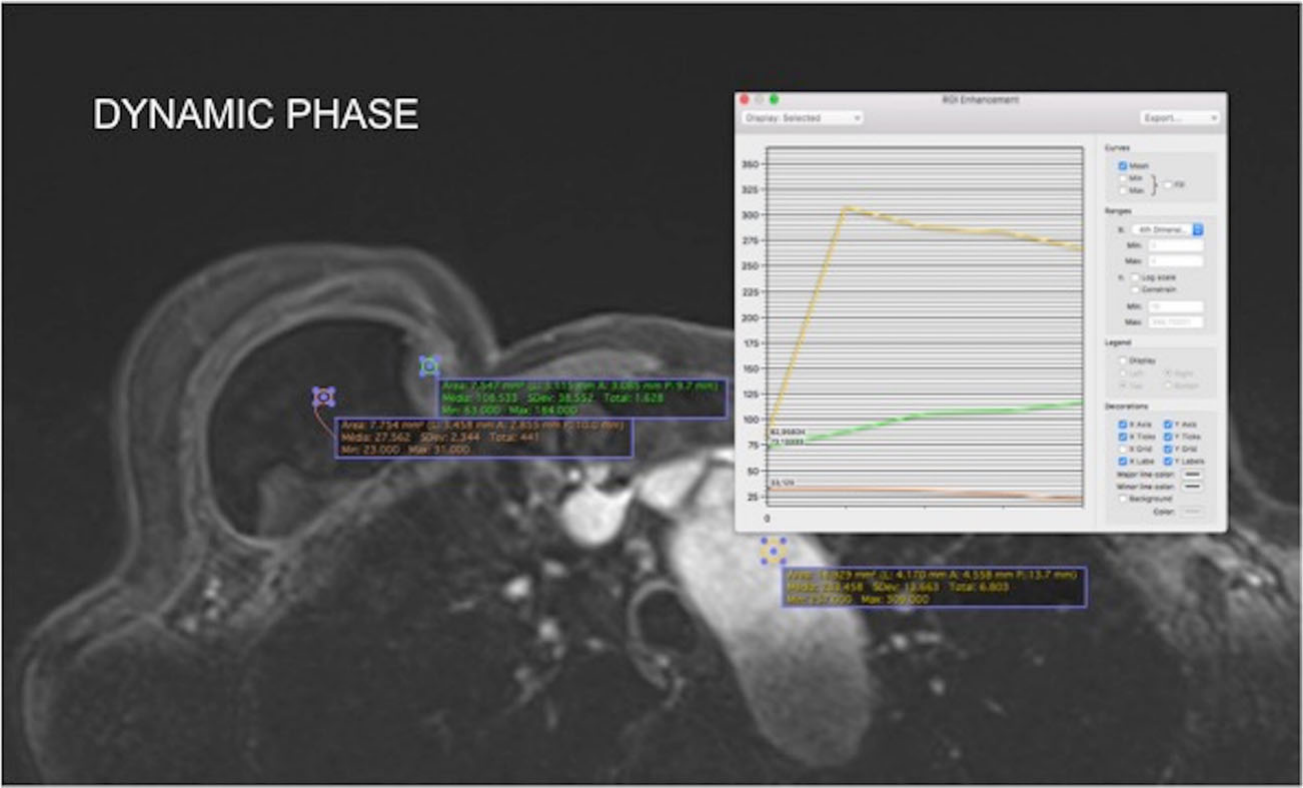
Enhancement pattern of the granuloma compared to the aortic arch and implant. The aortic arch presents a standard washout curve (type III) and the granuloma an ascending pattern (type I). The breast implant does not show contrast enhancement. A 57-year-old female underwent a mastectomy because of invasive carcinoma of the right breast with reconstruction of the breast using an implant for 5 years

The contrast enhancement of the mass is impaired by the presence of the barrier exerted by the intact fibrous capsule. Mass enhancement is progressive, with a type I dynamic curve pattern. Usually nodular areas of greater vascularisation appear within the mass. Without the late phase sequences after the use of contrast media, at least 4 min, differentiation from intracapsular haematoma is difficult (Fig. 7).

### Associated findings

**Fig. 8:**
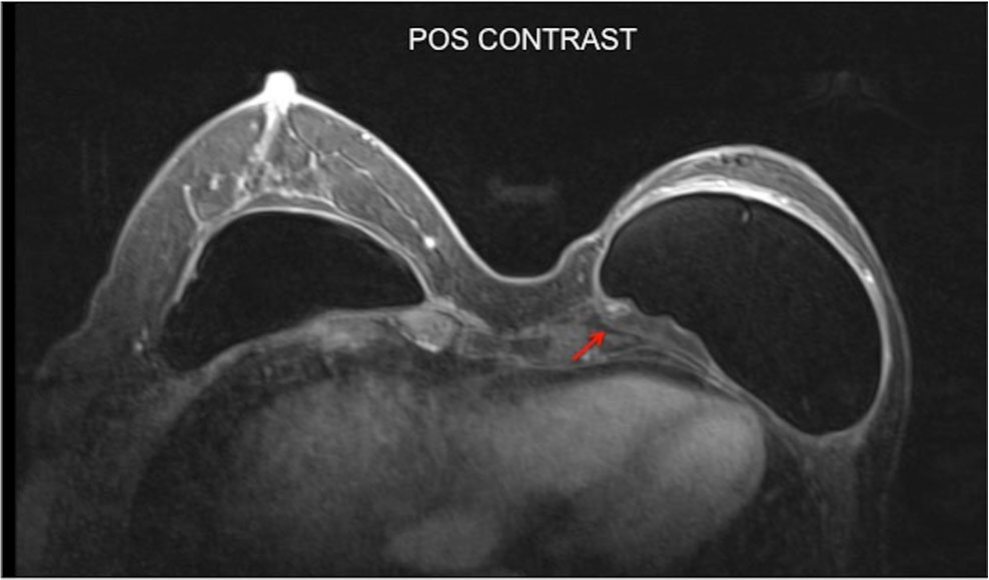
A 35-year-old female patient underwent mastectomy 3 years after invasive breast carcinoma. Granuloma is observed with heterogeneous enhancement at the posterior aspect of the implant (red arrow). Thickening and contraction of the fibrous capsule are associated in the anterior aspect, compatible with capsule contracture

Intracapsular haematoma/seroma may be associated with the mass. It is characterised by fluid collection that does not enhance with the contrast media (Fig. 7). The capsular contracture is determined by thickening of the fibrous capsule associated with an increase of the anteroposterior diameter. Enhancement of the fibrous capsule may be present to different degrees (Fig. 8).

## Imaging of granuloma in patients with intracapsular implant rupture

For comparative purposes, we describe a case of intracapsular breast implant rupture associated with granuloma of the fibrous capsule.

**Fig. 9:**
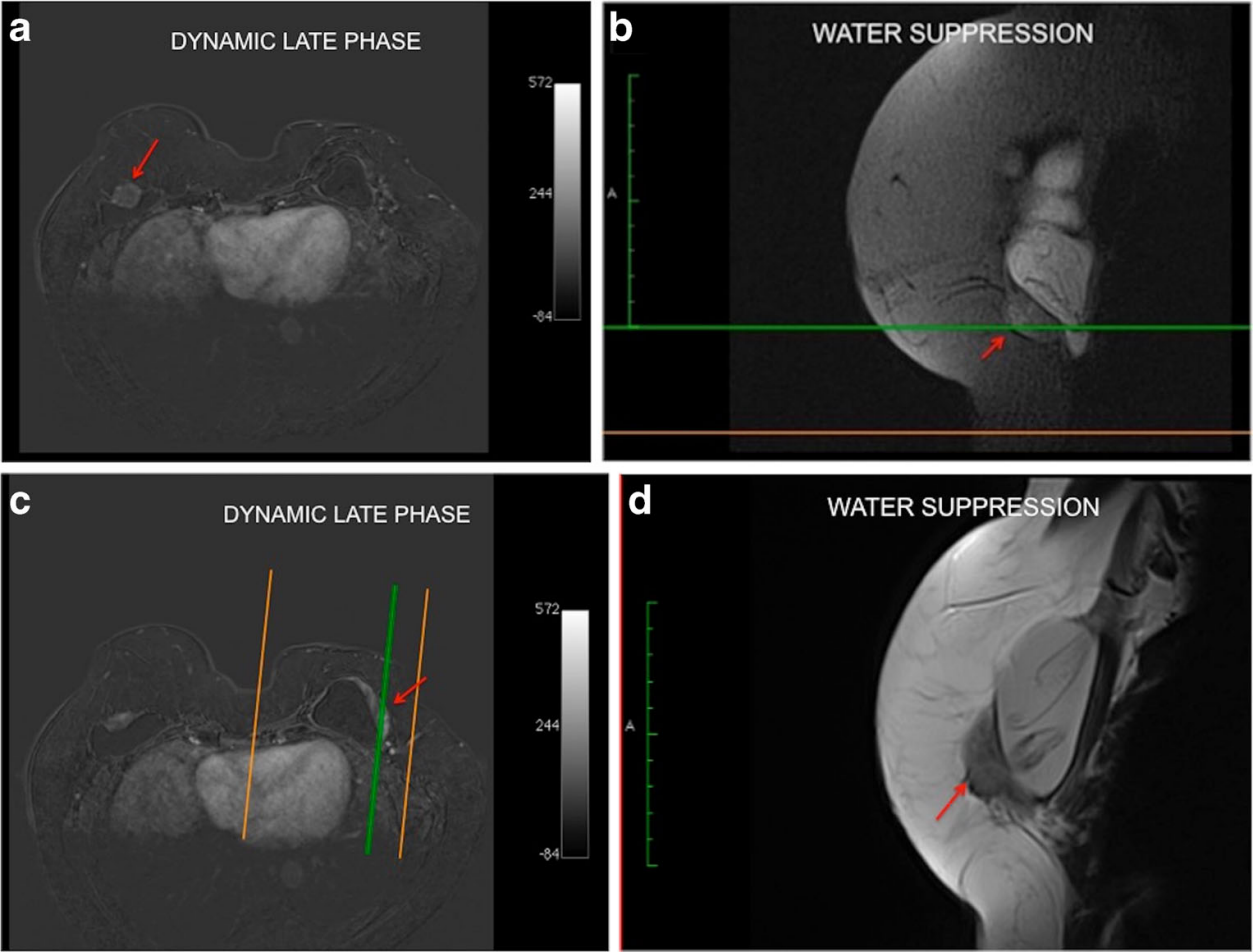
A 61-year-old patient underwent two breast implant replacement surgeries for contracture; the last one was performed 4 years ago (**a**, **b**, **c**, **d**). The post-contrast sequences present intracapsular masses with contrast enhancement in correlation with water suppression for the right breast (**a**, **b**) and for the left breast (**c**, **d**). Sequences with water suppression demonstrate intracapsular rupture of the implants (**b**, **d**)

The signs and symptoms are very similar to those found in silicone bleeding; however, the integrity of the implant elastomer is not maintained. The enhancement pattern and morphology of the granuloma are identical to those found when associated with intact implants since these patients may present the SIIS (Fig. 9).

## SIGBIC after implant removal and capsulectomy

**Fig. 10:**
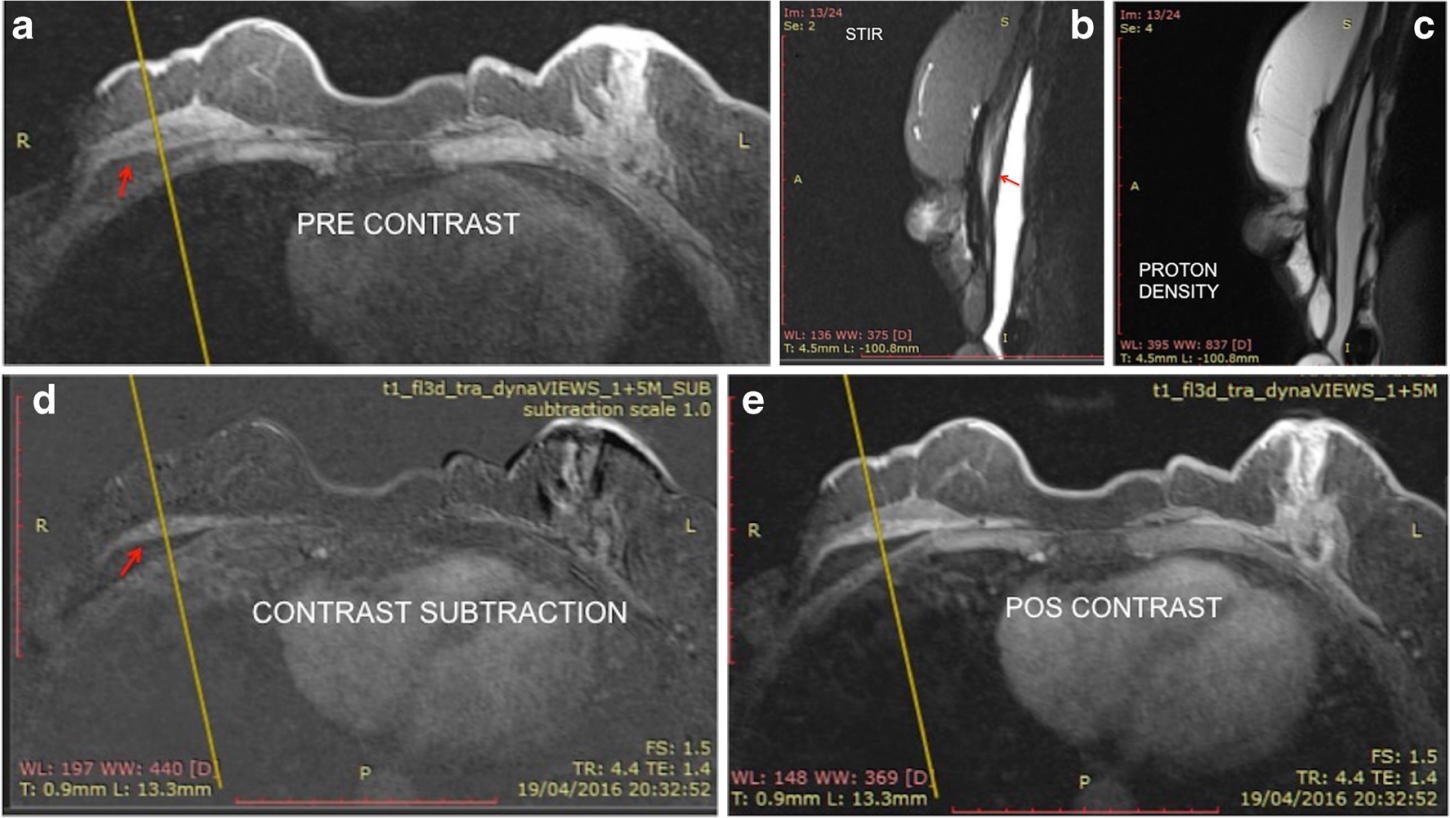
A 43-year-old HIV-positive patient with a history of rejection of two breast implants (**a**, **b**, **c**, **d**, **e**). Pre-contrast sequence presents collection in the implant site (**a**). Sequences of proton density and STIR show signs of thickening of the fibrous capsule (**b**, **c**). Post-contrast sequences present residual fibrous capsule granuloma (**d**, **e**)

The treatment of SIGBIC consists of implant removal and capsulectomy. If there is a remnant of the fibrous capsule in the implant site, fluid collection can be observed at the surgical site. It is recommended not to replace the silicone implant with another with the same content (Fig. 10).

It is important to emphasise that percutaneous diagnostic biopsies should be avoided in these cases. Performing the biopsy breaks the fibrous capsule barrier and exposes the rest of the body to the SIGBIC.

Preliminarily, we observed that some patients who undergo breast implant replacement because of capsule contracture and who in retrospective analysis have signals of SIGBIC at the pre-operative MRI scan tend to present early post-operative complications such as capsule contracture and intracapsular seroma.

We are carrying out a prospective study to evaluate the relationship between early contractures after implant replacement and SIGBIC.

## Patient management and follow-up

The medical management adopted for the SIGBIC (surgery or follow-up) in this pictorial essay was determined by the mastologists responsible for each patient.

Of the eight patients described, six chose surgical removal of the implant with capsulectomy. At histology, all six of these patients presented foreign body granuloma (silicone-content particles) with histological elements similar to those described by Cohen et al.

In the surgical specimen, SIGBIC presented as focal hardened areas at the fibrous capsule in the topography corresponding to the MRI findings. None of the silicone implants showed signs of rupture.

Two patients opted to have MRI scan follow-up despite being informed of the presence of SIGBIC. Despite the recommendation of implant removal and capsulectomy, half-yearly follow-up was suggested for these two cases.

As described, it is very important for the radiologist to recognise the characteristics of the SIGBIC to perform early diagnosis of this pathology. The diagnosis is relatively easy; however it requires using contrast medium where the lesions will be better evidenced in the late dynamic phases. We speculate that SIGBIC, when it overcomes the fibrous capsule, may determine a systemic autoimmune reaction that triggers a manifestation similar to anaplastic large cell lymphoma. Further studies should be performed to determine the association between SIGBIC and ALCL.

## Abbreviations

SIGBIC: Silicone-induced breast granuloma
ALCL: Anaplastic large cell lymphoma
ISAP: International Society of Aesthetic Surgery
FDA: Federal Drug Administration
MRI: Magnetic resonance imaging
TGF- β: Tumour growth factor β-1
ROS: Reactive of oxygen species
CBR: Colégio Brasileiro de Radiologia
